# The Neuroameliorative Effects of Enzymatically Modified Isoquercitrin and Sodium R-lipoate on the Rotenone rat Model of Parkinson’s Disease

**DOI:** 10.1007/s11064-026-04715-9

**Published:** 2026-03-25

**Authors:** Imam Hassouna, Omar H. Hassanein, Ibrahim A. El-Elaimy, Hany M. Ibrahim

**Affiliations:** Physiology and Immunology Unit, Zoology Department, Faculty of Science, Menoufia University, Shebin El-Kom, Egypt

**Keywords:** Rotenone, Enzymatically modified isoquercitrin, Sodium R-lipoate, Parkinson’s disease, Neuroinflammation

## Abstract

Parkinson’s disease (PD) is a neurodegenerative condition distinguished by both motor and non-motor signs. Currently, therapeutic interventions effectively mitigate motor symptoms; however, they fail to impede the progression of neurodegeneration. Enzymatically modified isoquercitrin (EMIQ) is a variant of isoquercitrin with enhanced bioavailability and potentially greater health benefits. Sodium R-Lipoate (NaRLA) is a modified form of lipoic acid with improved stability and efficacy. The current work assessed EMIQ and NaRLA neuroameliorative properties in a rotenone (ROT) model of PD in rats. The study employed open field and hanging tests to evaluate motor effects, and used dopamine (DA) estimation and tyrosine hydroxylase (TH) immunoreactivity to confirm motor abnormalities and neurodegeneration. Furthermore, neuroinflammation was evaluated through ELISA of proinflammatory cytokines (IL-1β, IL-6), expression of Toll-like receptor 4 (TLR4) via qRT-PCR, and immunoreactivity of ionized calcium-binding adaptor molecule 1 (IBA1). The findings indicated that the use of EMIQ and NaRLA with ROT reduced the neuroinflammation via decreasing: the expression level of TLR4, IL-1β and IL-6 levels, and the number of IBA1^+^ microglial cells. Moreover, EMIQ and NaRLA ameliorated motor impairments induced with ROT through the enhancement of rats’ behavior, increasing DA concentration and TH immunoreactivity in the striatum. In the light of the findings of the current investigation, EMIQ and NaRLA exerted neuroameliorative effects against the ROT‐induced PD model. These results provide support for the potential impact of EMIQ and NaRLA in the treatment of PD and other diseases related to mitochondrial dysfunction and neuroinflammation.

**Supplementary Information:**

The online version contains supplementary material available at 10.1007/s11064-026-04715-9.

## Introduction

Parkinson’s disease (PD) is a neurological disease that is more prevalent in the elderly; however, recent epidemiological data show a rising incidence of early-onset cases (21–50 years), highlighting the disease’s growing impact on younger age groups [1]. Dopaminergic neuron degeneration in the substantia nigra pars compacta (SNpc), which results in a dopamine (DA) deficiency in the striatum, is the primary etiology of the disease [2]. PD is distinguished by an assortment of motor as well as non-motor symptoms. The former includes bradykinesia (movement slowness), resting tremor, postural instability, rigidity, and gait imbalance, while the latter includes cognitive and mental abnormalities, autonomic dysfunction, sensory impairment, and sleep disturbances [3, 4]. The precise mechanism of PD remains incompletely elucidated. PD pathogenesis involves several pathways, including inflammation [5], mitochondrial dysfunction [6], apoptosis [7], and abnormal redox status [8].

Neuroinflammation substantially impacts the onset and development of degenerative neuropathies such as PD, Huntington’s disease, and Alzheimer’s disease [9, 10]. Microglia, the innate immune cells of the brain, are easily stimulated when brain damage or neurodegenerative processes occur. Subsequently, these cells rapidly multiply, undergo hypertrophy, and secrete proinflammatory cytokines and neurotoxic substances [11, 12]. The activation of innate immune receptors, particularly Toll-like receptors (TLRs), is a key driver of neuroinflammatory processes implicated in PD. Beyond their classical role in detecting pathogens and central nervous system injury [13], TLRs initiate intracellular signaling cascades that induce the expression of pro-inflammatory cytokines and other immune mediators [14]. In the SNpc, TLR activation within microglia acts as a critical upstream event that amplifies neuroinflammation, promotes oxidative stress, and contributes directly to the degeneration of dopaminergic neurons [15]. This mechanistic link highlights the central role of TLR signaling in the progressive dopaminergic neuronal loss characteristic of PD.

Animal models are crucial tools for studying PD, as they enable the replication of key pathological and behavioral features observed in the human condition. Among these models, rotenone (ROT), an inhibitor of mitochondrial complex I widely used in agriculture as an herbicide and pesticide, has been extensively employed to induce PD-like symptoms in animals [16]. Rats treated with ROT exhibit several hallmark features reminiscent of sporadic PD, including motor impairments such as bradykinesia and reduced locomotor activity, neuroinflammation, activation of microglial cells, decreased levels of endogenous antioxidants, mitochondrial dysfunction, neuronal degeneration and increasing endoplasmic reticulum stress markers [17–20]. Due to these characteristics, the ROT-treated animal model serves as a valuable platform for investigating novel therapeutic strategies targeting multiple pathways implicated in PD pathogenesis.

Although the current treatments are effective at managing motor symptoms, they do not prevent neurodegeneration, disease progression, or disability escalation. New treatments and approaches are needed to slow down or stop the progression of the disease [21]. Enzymatically modified isoquercitrin compound (EMIQ) is formed through trans-glycosylation of isoquercitrin with malto-oligosaccharides using cyclodextrin glucanotransferase [22]. Although EMIQ is metabolized similarly to rutin and is absorbed as quercetin, its bioavailability is approximately seventeen times that of quercetin [23]. Although isoquercitrin has demonstrated neuroprotective effects in the 1-Methyl-4-phenyl-1,2,3,6-tetrahydropyridine (MPTP) model of PD, improving behavioral deficits, inhibiting dopaminergic neuron degeneration, and upregulating markers such as tyrosine hydroxylase (TH) and DA transporters [24], the pathophysiological mechanisms of the MPTP and ROT models differ substantially [25]. The ROT model replicates key features of sporadic PD, including mitochondrial complex I inhibition, oxidative stress, neuroinflammation, and progressive neuronal loss [26]. Preclinical studies on EMIQ have primarily focused on its anti-inflammatory and antioxidant properties; however, its efficacy against ROT-induced neurotoxicity, characterized by mitochondrial dysfunction and oxidative damage, remains underexplored. Therefore, evaluating EMIQ in the ROT model is essential to determine its therapeutic potential across diverse, pathologically relevant mechanisms of PD progression.

Sodium R-lipoate (NaRLA), the sodium salt of the biologically active R-enantiomer of α-lipoic acid (ALA), is an organosulfur compound naturally present in plants, animals, and humans [27]. Although ALA has demonstrated neuroprotective effects in PD models, its therapeutic application is limited by extensive first-pass hepatic metabolism and low systemic bioavailability [28]. Converting ALA into its sodium salt form improves its aqueous solubility and chemical stability [29], which is expected to enhance intestinal absorption and potentially increase tissue and brain exposure. Although the specific anti-inflammatory mechanisms of NaRLA have not yet been fully elucidated, several well-established properties of its parent compound provide a strong rationale for its investigation in PD. These include its ability to support mitochondrial redox homeostasis and sustain intracellular glutathione levels. Previous studies demonstrated that ALA enhances antioxidant defenses via activation of the PI3K/AKT pathway in ROT-induced PD models [30] and mitigates ferroptosis by modulating iron metabolism and upregulating glutathione peroxidase 4 expression [31]. Together, these pharmacokinetic improvements and mechanistic insights provide reasonable justification for investigating NaRLA in a ROT-induced PD model, despite the absence of a fully characterized mechanism.

In this study, we investigated how ROT exposure alters the inflammatory response in four key brain regions implicated in PD: the cortex, hippocampus, striatum, and substantia nigra. The objectives of this approach were threefold: (1) to determine whether the striatum exhibits distinct vulnerabilities compared with the cortex and hippocampus; (2) to obtain a comprehensive understanding of the region-specific inflammatory responses associated with PD; and (3) to evaluate the neuroameliorative effects of EMIQ and NaRLA in the ROT-induced PD rat model.

## Materials and Methods

### Animals

Adult Wistar male rats, with an average weight 200-250 g were bought from the holding company for biological products and vaccines (VACSERA), Cairo, Egypt. Animals were housed in standard polypropylene cages (4 rats/cage) with stainless-steel wire lids. Rats were maintained under controlled environmental conditions (25 ± 1 °C, 12:12 h light/dark cycle). Rats were provided a standard laboratory chow diet (Meladco Feed Company, Obour City, Cairo, Egypt) containing approximately 20% protein, 5% fat, 5% fiber, and a balanced vitamin–mineral premix, with clean water available ad libitum. The animals underwent a minimum of ten days of acclimation to laboratory conditions preceding the initiation of the experiment. All procedures were performed in accordance with the Guide for the Care and Use of Laboratory Animals (8th edition, 2011) [32] and were approved by the Institutional Animal Ethical Committee of Menoufia University, Egypt (Approval ID: MUFS/F/PH/1/22).

## Chemicals and Drugs

ROT (≥ 95%; Cat#: R8875) and DMSO (≥ 99.9%; Cat#: 472,301) were acquired from the Sigma-Aldrich Company located in Missouri, USA. EMIQ was provided by the Natural Factors Company in Coquitlam, Canada, whereas NaRLA was provided by the Nature’s Answer Company in New York, USA. Formalin (Code: F/1501/PB17) was obtained from Fisher Scientific (Loughborough, UK). All other chemical compounds were of the highest available analytical grade and purity.

## Experimental Design

**Fig. 1:**
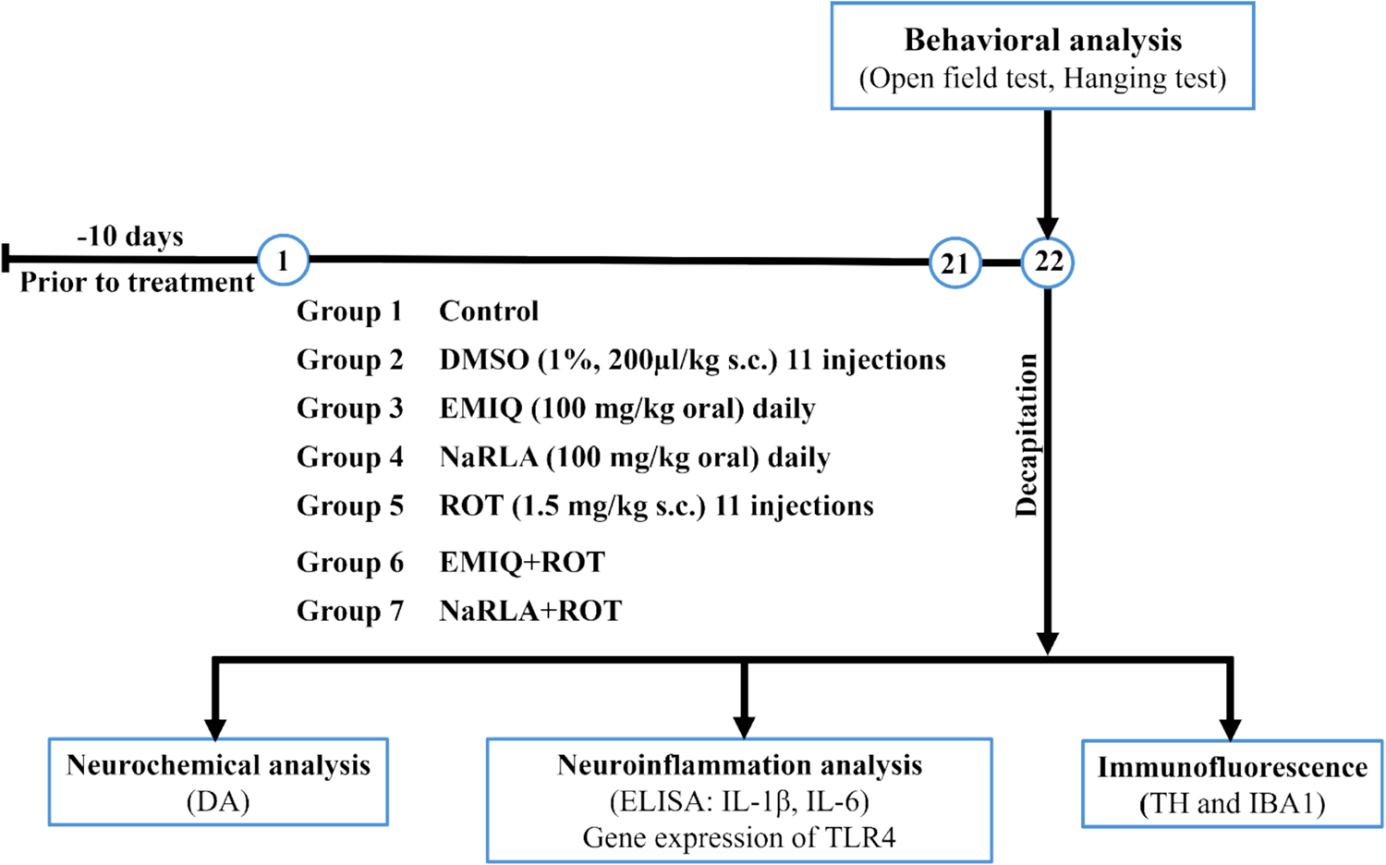
Experimental design & measured parameters. Behavioral tests were carried out on day 22. Immediately after testing, rats were deeply anesthetized with isoflurane (5%) and sacrificed by decapitation for tissue collection. DA; dopamine, ELISA; Enzyme-linked immunosorbent assay, IL-1β; interleukin-1 beta, IL-6; interleukin-6, TLR4; Toll-like receptor 4, TH; tyrosine hydroxylase, IBA1; ionized calcium-binding adaptor molecule 1, s.c.; subcutaneous, DMSO; dimethyl sulfoxide, EMIQ; enzymatically modified isoquercitrin, NaRLA; sodium R-lipoate, ROT; rotenone

One hundred twelve rats were randomly allocated into seven groups, each consisting of sixteen rats. Group (1): normal control group. Group (2): rats received eleven injections of 1% dimethyl sulfoxide (DMSO, 200 µl/Kg) subcutaneous (s.c.) every other day for a period of twenty-one days. In groups 3 and 4, rats were orally administered 100 mg/kg of EMIQ and NaRLA respectively every day for 21 days [33, 34]. Group (5): rats were injected every other day for 21 days with ROT dissolved in 1% DMSO at a dose of 1.5 mg/kg s.c. [35]. Group (6): rats were administered ROT and EMIQ; and on days when ROT was injected, EMIQ was administered one hour prior to the ROT injection. Group (7): rats received ROT and NaRLA as previously explained, NaRLA was administered one hour preceding the ROT injection. At the end of the treatment period, behavioral assessments (open field and hanging tests) were performed on day 22. Immediately after completing the behavioral testing, all rats were deeply anesthetized with isoflurane (5%) until loss of the pedal withdrawal reflex, followed by decapitation, and immediately brain removal from the skull for biochemical, qRT-PCR, and immunofluorescence analyses (Fig. 1).

## Behavioral Tests

The behavioral evaluation was conducted during the light phase, on day 22. The rats had an initial screening through the open-field test, followed by the hanging experiment, with a two-hour interval between them. To ensure uniformity and maintain equal statistical power across all groups during behavioral testing, behavioral analyses were conducted on 10 animals per group, representing those that completed the full experimental protocol.

## Open Field Test

An open field test is utilized to evaluate the overall locomotion activity and the degree of environmental exploration. A box (made of wood) measuring 80 by 80 cm, with walls that are 40 cm in height, was used as equipment for the open field. A black ground was marked with white lines that split the field into grid zones (5 × 5) that were each equal in size, measuring 16 × 16 cm. Each rat was given three trials before the experiment started to ensure reliable reflection of the true effects of the experimental treatment as opposed to variability from first-time exposure. Following a three-minute video recording period for every rat, the videos were examined to ascertain the subsequent parameters: rearing frequency (the frequency at which the animals assumed an upright position using their hind paws), immobility time (the time duration in which the animals remained motionless), and locomotion frequency (the number of times the animals passed from one quadrant to another) [36, 37].

## Hanging Test

The hanging test is a method used to monitor neuromuscular strength and motor function. The apparatus comprises of a rectangular box with a horizontal 12 mm diameter metal rod situated at an elevation of 30 cm above the ground. Twenty-four hours subsequent to the final administration of ROT, after finishing the open field test, each rat was suspended from the metal rod to measure the duration till it touched the ground. The rats’ performance was replicated 3 times, with each interval between each test, lasting 1 min. Subsequently, the hanging time has been documented [38].

## Sampling and Brain Processing

Following the evaluation of motor functions, each group of animals was divided randomly into three subsets and euthanized under isoflurane anesthesia (5%). Following dissection, brains were washed with ice-cold saline and processed for various analyses. For immunofluorescence, a subset of four brains was fixed in 10% neutral buffered formalin. The striata from the remaining animals were promptly dissected and preserved at -80 °C temperature and then divided into two subsets for the measurement of various biomarkers. One subset (n = 6 striata) was used for measuring Toll-like receptor 4 (TLR4) with quantitative real-time PCR. The other subset (n = 6 striata) was homogenized using cold phosphate-buffered saline (PBS, pH 7.4) and used to measure DA, interleukin-6 (IL-6), and interleukin-1 beta (IL-1β).

## Biochemical Measurements

### Estimation of Striatal DA

DA concentration in rat striatum was determined using MyBioSource DA ELISA Kit (San Diego, California, USA), Cat#: MBS725908 conferring to the manufactural prescribed methods.

## Estimation of Proinflammatory Cytokines (IL-1β and IL-6)

The striatal IL-1β level was determined using the ELISA kit (Cat#: MBS825017) obtained from MyBioSource (San Diego, California, USA), according to manufacturers’ prescripts. IL-6 was determined in the striatal tissue according to the manufactural instructions using ELISA kit (Cat#: NBP1-92,697) from Novus Biologicals (Centennial, Colorado, USA). The total protein levels in the striatal homogenate were assessed using MyBioSource Colorimetric Assay Kit (BCA Method), Cat#: MBS2540455. Concentration of cytokines were expressed as pg/mg tissue protein.

## Quantitative Real-Time PCR (qRT-PCR) Analysis

The extraction of total RNA from the final sample was done via a RNeasy Plus Universal Mini Kit (ID: 73,404: Qiagen, Hilden, located in Germany), as directed by the supplier. Briefly, RNA was extracted from each striatal tissue to yield 40 µL of RNA solution. The RNA’s quality was assessed using a Nanodrop Spectrophotometer (ratio of A260/280). The purified RNA was promptly utilized for cDNA synthesis. The ReverAid RT Kit obtained from ThermoFisher Scientific situated in Waltham in USA, was employed to do reverse transcription of 5 µL of RNA, following the manufacturer’s prescribed methods. The Rotor Gene Q platform was used to perform real-time PCR Amplification in order to measure mRNA expression levels. Specific primer sets for TLR4, and GAPDH were utilized for this purpose. The primer sequences for TLR4 and GAPDH are listed in Table 1. As a control, the housekeeping GAPDH was employed. The cDNA was combined with Maxima SYBR Green Master Mix (ThermoFisher Scientific, Waltham, USA) in a final volume of 20 µl. Real-time PCR reactions were performed at a temperature of 95 °C for a duration of 5 min, followed by 40 cycles of 15 s at 95 °C and 30 s at 60 °C. The Ct values normalized to the values of the housekeeping gene. To quantitatively evaluate the relative target expression levels, the 2^−ΔΔCt^ method was utilized [39].

**Table 1:** Primer sequences for TLR4 and GAPDH

Gene	TLR4	GAPDH
Forward primer	5′-CATGACATCCCTTATTCAACCAAG-3′	5′-CCTTCATTGACCTCAACTAC-3′
Reverse primer	5′-GCCATGCCTTGTCTTCAATTG-3′	5′-TTCACACCCATCACAAAC-3′
Accession No	NM_019178.2	XM_063288584.1

## Immunofluorescence

In our study, some precautions have been taken to ensure adequate fixation and maintain tissue integrity. These included rapid brain removal from the skull, immediate immersion of tissues in 10% neutral buffered formalin following dissection, dividing the brains into hemispheres, and maintaining a fixation period of 72 h to ensure complete cross-linking. Brains were subjected to sequential processing in varying grades of ethanol, and clearing in xylene, followed by infiltration, as well as embedding into paraffin. Rotatory microtome slices of sagittal brain Sections (5 μm thick) were prepared. The sagittal brain levels were identified according to Figs. 79–85 of Paxinos and Watson (2007) [40]. Paraffin-impeded sections were deparaffinized in xylene followed by a serial dilution of ethyl alcohol till complete hydration. Sections underwent treatment with citrate buffer pH 6.0 for antigen retrieval. Afterward, they were permeabilized and subsequently blocked with 5% normal horse serum, 0.3% Triton-X in PBS for one hour at room temperature. Sections were incubated with primary antibodies: polyclonal rabbit anti-TH (1:1000; Ab152; Chemicon) and monoclonal chicken anti-IBA1 (1:1000; 234009; SySy Göttingen) overnight at 4 °C. The sections were incubated with the appropriate secondary antibodies after being washed with PBST (Goat anti-chicken IgY Dylight 650; Donkey anti-rabbit IgG Alexa Fluor555, all from Invitrogen) for 2 hours with a dilution of 1:1000. After washing, DAPI was used for counter staining, dried overnight and coverslipped by Aqua Poly/Mount. Images were obtained using microscope (Zeiss Axioscan: Z1) and analyzed using Fiji and ZEN blue 3.5 software to count the total number of IBA1^+^ microglial cells in cortex (primary motor cortex M1), hippocampus, striatum and SNpc regions. For quantitative analysis, four sections obtained from each brain area (cortex, hippocampus, striatum, and SNpc) were analyzed in each animal. The area of brain regions was determined for each section. Cell density was obtained by dividing the number of double-labeled cells (IBA1^+^/DAPI^+^) per section by the area of brain region in mm^2^. Fiji also used to estimate the % area of TH expression in striatum and SNpc as well as for the measurement of the area of microglial cell soma. All immunofluorescence staining procedures were conducted by a well-trained investigator blinded to group allocation. To ensure unbiased data collection, cell counting and quantification were performed in a blinded manner, with the observer unaware of all treatment conditions.

## Statistical Analysis

The statistical analyses were accomplished utilizing Version 10 of GraphPad Prism. The resulting data were then presented as mean ± standard error of the mean (SEM). Normality was assessed using the Shapiro–Wilk test, and homogeneity of variances was evaluated using the Brown–Forsythe test in GraphPad Prism. The assumptions for ANOVA were satisfied. The one-way ANOVA test was utilized to conduct analysis of variance among the groups. Subsequently, Tukey’s post hoc test was employed to evaluate group differences, and statistical significance was denoted as follows: *P < 0.05, **P < 0.01, ***P < 0.001, and ****P < 0.0001.

## Results

### Effect of EMIQ and NaRLA on Motor Performance

**Fig. 2:**
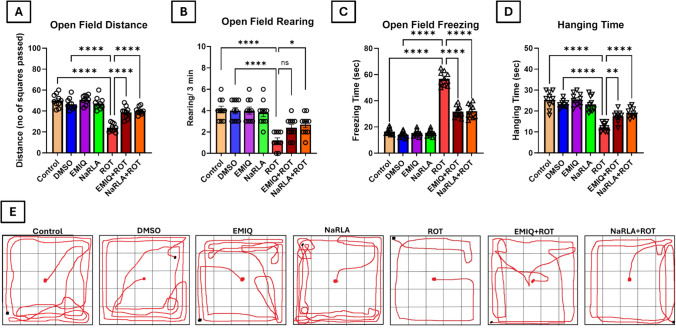
The behavioral effects of EMIQ and NaRLA on rats subjected to ROT in both open field and hanging experiments. The charts illustrate the distance covered (**A**), the frequency of rearing (**B**), the duration of freezing (immobility) (**C**), the duration of hanging (**D**), and representative track plots illustrating the locomotor activity of rats during the open field test (**E**). Data are displayed as mean ± SEM, with *n* = 10. Group comparisons were analyzed using ANOVA, followed by Tukey’s multiple comparisons test. Significant levels are denoted through the following order: *P* < 0.05 (*), *P* < 0.01 (**), *P* < 0.0001 (****); ns indicates no significant difference

Treatment with DMSO, EMIQ, or NaRLA did not result in statistically significant alterations in open field or hanging test outcomes relative to the normal control group. Specifically, distance traveled (squares traversed by rat), rearing behavior (the frequency with which the animals assumed a hind paw position), and freezing time (duration of immobility during the test in seconds) were comparable among the normal control, DMSO, EMIQ, and NaRLA groups (50.00 ± 1.95, 46.50 ± 1.71, 50.80 ± 1.35, and 47.20 ± 1.83 squares; 4.10 ± 0.31, 4.00 ± 0.30, 4.00 ± 0.33, and 3.80 ± 0.36 number of events/3 min; and 16.50 ± 0.81, 14.20 ± 0.73, 15.40 ± 0.73, and 15.50 ± 0.75 s, respectively). Representative total distance traveled, rearing behavior, freezing time, and tracking paths are shown in Fig. 2A, B, C & E, respectively. However, animals treated with ROT alone showed a significant decrease in distance and rearing (24.00 ± 1.23 and 1.20 ± 0.25, respectively), while showing a significant increase in freezing time (56.80 ± 1.66) in comparison with normal control and DMSO groups. Compared to ROT-injected rats, EMIQ + ROT group exhibited a significant increase in distance and a non-significant rise in rearing behavior (38.70 ± 1.74 and 2.40 ± 0.31, respectively), associated with a significant reduction in freezing time (31.60 ± 1.38). Animals treated with NaRLA + ROT showed a significant increase in distance and rearing (40.00 ± 1.07 and 2.70 ± 0.30), accompanied by a notable reduction in freezing time (32.10 ± 1.64) in comparison to rats injected with ROT. In the hanging test, the ROT group demonstrated a statistically substantial reduction in hanging time (12.20 ± 0.63 s) in comparison to both the normal control and DMSO groups (25.30 ± 1.29 and 23.30 ± 0.56, respectively). Additionally, the groups treated with EMIQ + ROT or NaRLA + ROT showed significant elevation in hanging time (17.50 ± 0.83 and 19.20 ± 0.71, respectively) compared to the ROT group (Fig. 2D).

## Effect of EMIQ and NaRLA on Dopaminergic Neuronal Integrity

**Fig. 3:**
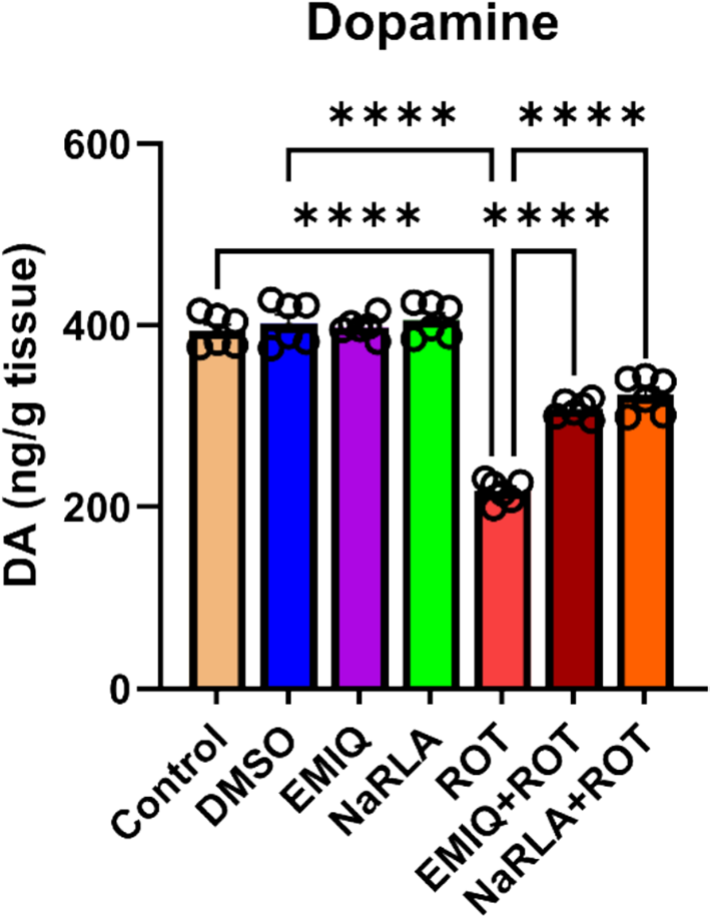
The impact of EMIQ and NaRLA administration on striatal dopamine levels in rats treated with rotenone. n = 6, Data are expressed as mean ± SEM, analyzed using one-way ANOVA with Tukey’s post hoc test for multiple comparisons, ****P < 0.0001

**Fig. 4:**
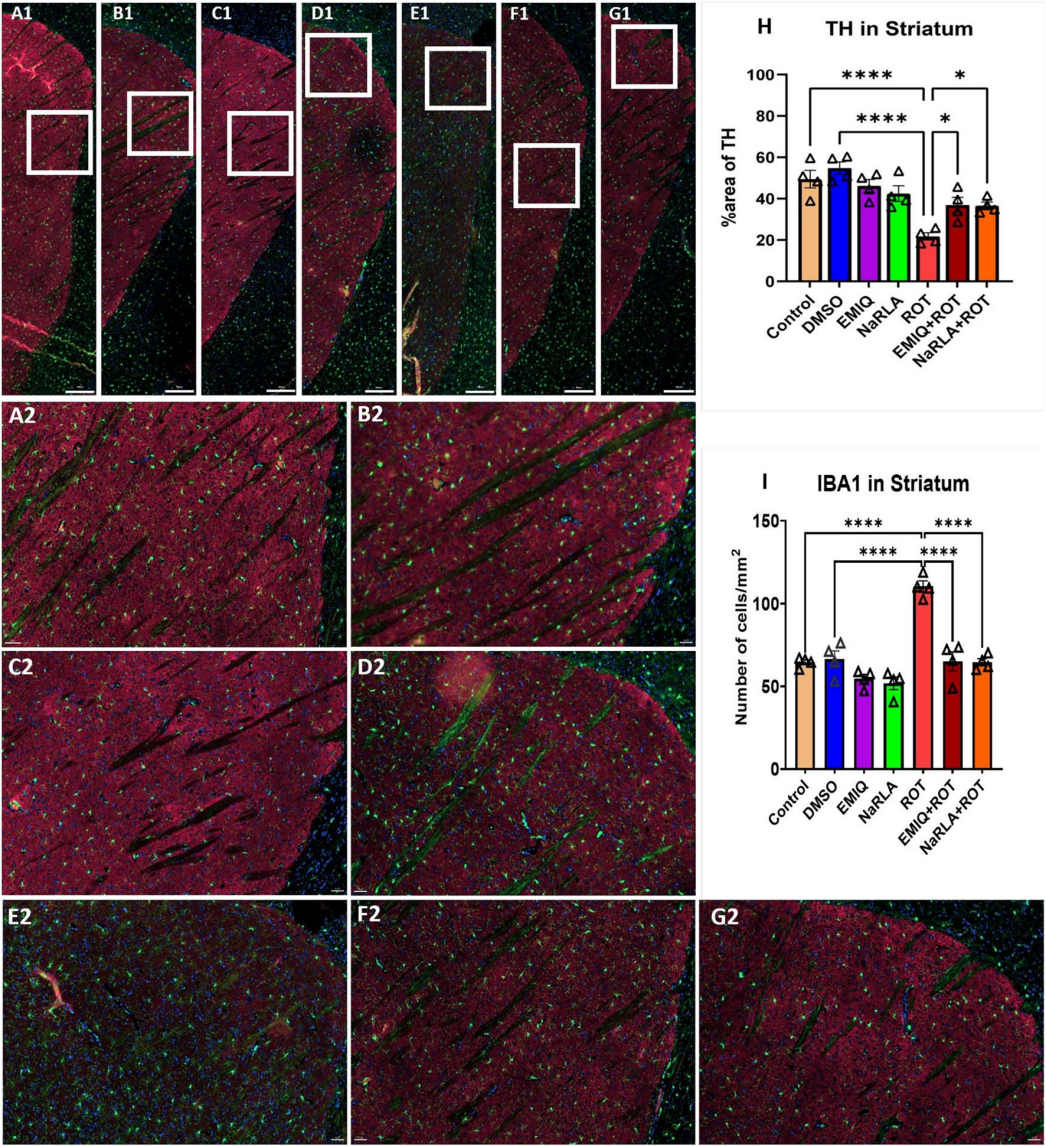
The effect of EMIQ and NaRLA on the TH and IBA1 immunoreactivity in striatum of ROT-treated rats. Representative immunofluorescence photomicrographs showing TH and IBA1 immunoreactivities in the striatum of the normal control group (**A1**), DMSO group (**B1**), EMIQ group (**C1**), NaRLA group (**D1**), ROT group (**E1**), EMIQ + ROT group (**F1**), and NaRLA + ROT group (**G1**). Scale bars: A1-G1 = 300 µm; A2-G2 = 50 µm. The squares in the overview are represented by the higher magnification images A2-G2. Quantitative analysis of the percentage area of TH immunostaining (**H**) and the number of IBA1⁺ microglia in the striatum (**I**) is provided for all groups. Blue = DAPI, green = IBA1, and red = TH. n = 4 rats/group and four sections per animal, data are expressed as mean ± SEM. One-way ANOVA and subsequent post hoc Tukey’s test; *P < 0.05, ****P < 0.0001)

**Fig. 5:**
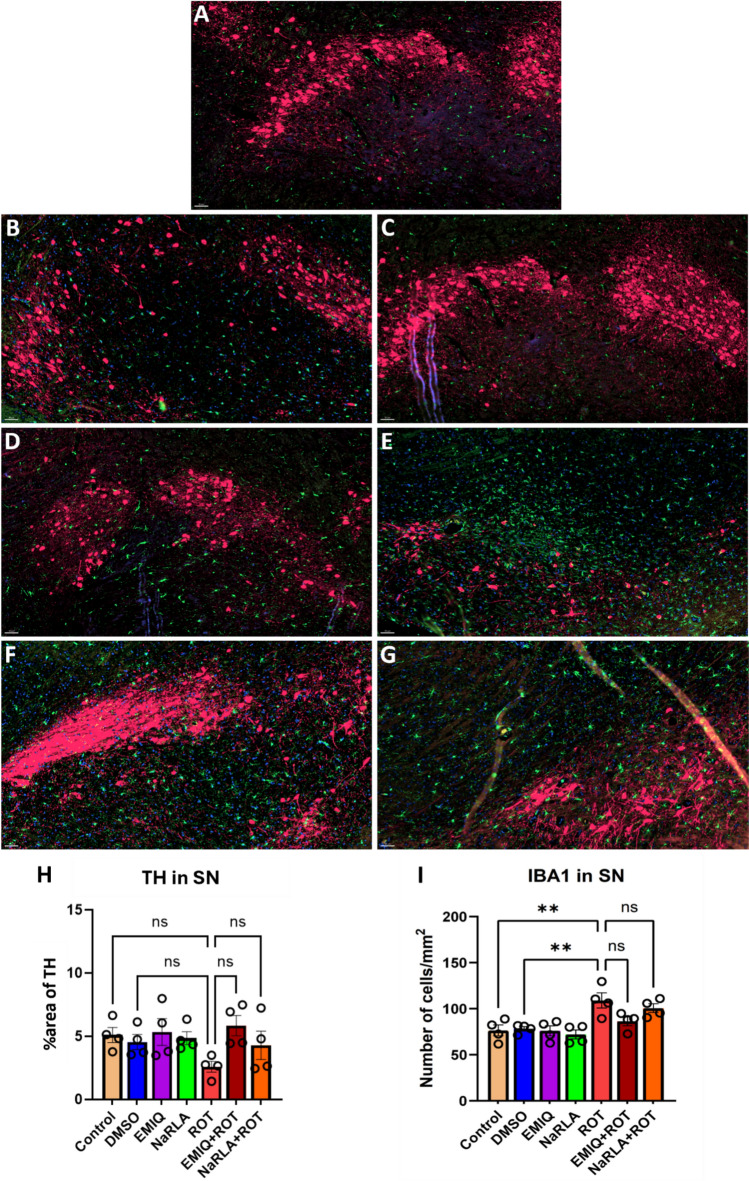
Effect of EMIQ and NaRLA on the immunoreactivity of TH and IBA1 microglia in the SNpc of rats-treated with ROT. Representative immunofluorescence photomicrographs of TH and IBA1 in the SNpc are shown for the normal control group (**A**), DMSO group (**B**), EMIQ group (**C**), NaRLA group (**D**), ROT group (**E**), EMIQ + ROT group (**F**) as well as NaRLA + ROT group (**G**). Scale bar = 50 µm. The percentage area of TH immunostaining within the SNpc (**H**) and the number of IBA1^+^ microglia (**I**) has been quantitatively analyzed for all groups. Blue = DAPI, green = IBA1, and red = TH. n = 4 rats/group and four sections per animal, data are presented as mean ± SEM. (one-way ANOVA and subsequent post hoc Tukey’s analysis; **P < 0.01, ns: no significance)

Tyrosine hydroxylase (TH) is the rate-limiting enzyme in the biosynthesis of DA, the neurotransmitter involved in motor brain functions and striatum is heavily involved in DA-related functions. To assess if EMIQ or NaRLA protected dopaminergic neurons from the deteriorating effect of ROT, we estimated alterations in striatal DA levels together with TH immunoreactivity within the striatum and SNpc. Rats in the DMSO, EMIQ, or NaRLA groups exhibited non-significant changes in DA concentration (402.67 ± 9.59, 398.33 ± 4.51, and 406.33 ± 7.53 ng/g tissue, respectively) compared to the normal control group (394.17 ± 7.28). The ROT group displayed a noteworthy reduction in DA levels (217.50 ± 5.06 ng/g tissue) compared to the normal control or DMSO groups. The concentration of DA in rats treated with EMIQ + ROT or NaRLA + ROT (307.83 ± 3.97 and 323.50 ± 8.31 ng/g tissue, respectively) was significantly higher than in rats injected with ROT alone (Fig. 3). Given that TH expression reflects dopaminergic neuronal integrity and DA biosynthetic capacity, changes in striatal DA levels were further supported by assessing TH immunoreactivity. Treatment with ROT produced a highly significant reduction of TH-positive fibers in the striatum (Fig. 4) and an obvious loss of TH-immunoreactive neurons in the SNpc (Fig. 5). Concurrent administration of EMIQ or NaRLA with ROT significantly attenuated the reduction in striatal TH immunoreactivity and DA levels compared with the ROT group. In the SNpc, both treatments produced a non-significant increase in TH-positive neurons compared with the ROT group, indicating a trend toward preservation of dopaminergic integrity (Fig. 5).

## Effect of EMIQ and NaRLA on Neuroinflammatory Parameters

**Fig. 6:**
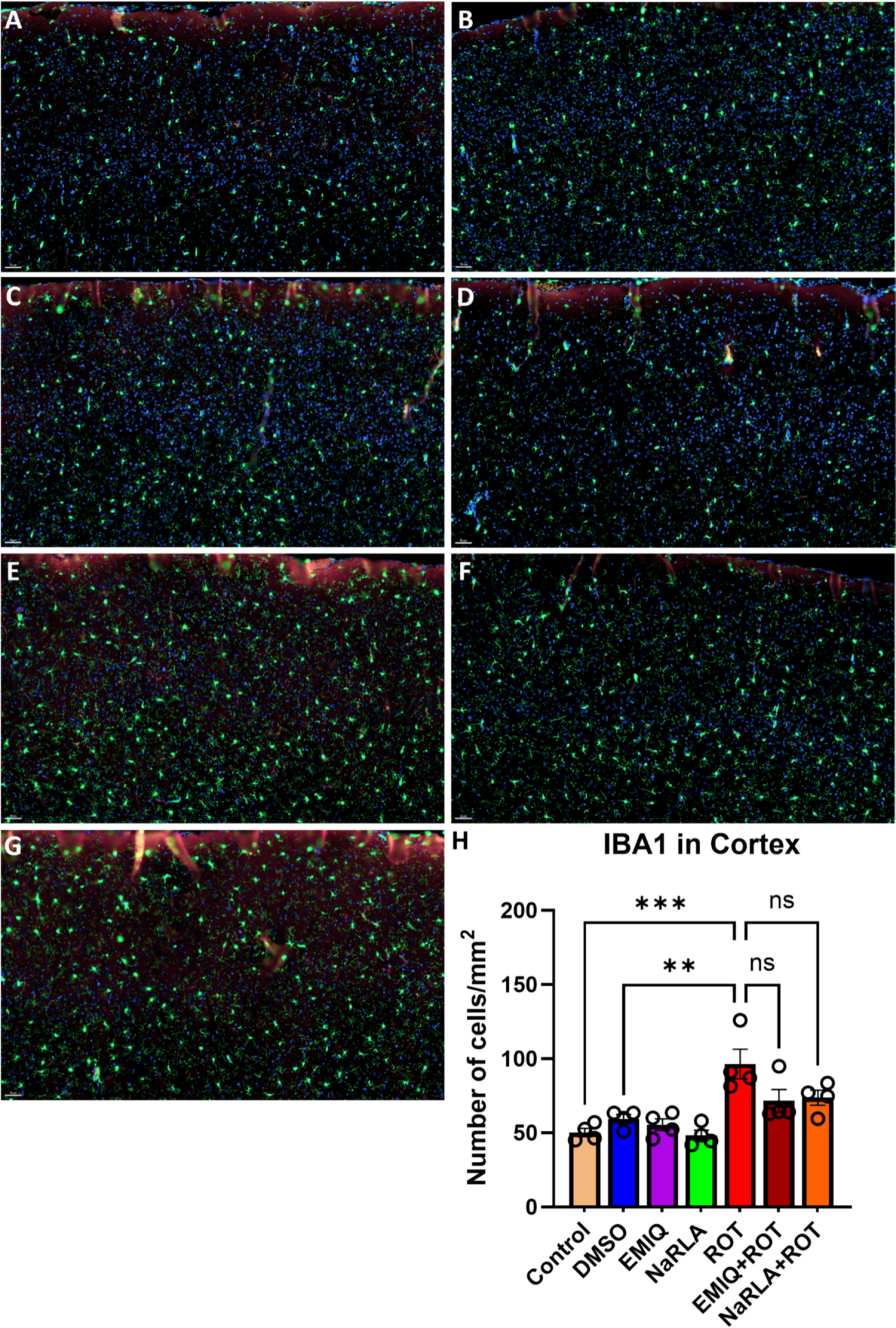
Effect of EMIQ and NaRLA on IBA1 positive microglia in the cortex of ROT-treated rats. Representative immunofluorescence photomicrographs of IBA1^+^ microglia in the cortex are shown for the normal control group (**A**), DMSO group (**B**), EMIQ group (**C**), NaRLA group (**D**), ROT group (**E**), EMIQ + ROT group (**F**) as well as NaRLA + ROT group (**G**). Scale bar = 50 µm. Quantitative analysis of the number of IBA1^+^ microglia in the cortex is provided for all groups (**H**). Blue = DAPI, green = IBA1. Data expressed as mean ± SEM (n = 4 rats per group and four sections per animal). (ANOVA with subsequent Tukey’s post hoc analysis; **P < 0.01, ***P < 0.001, ns: no significance)

**Fig. 7:**
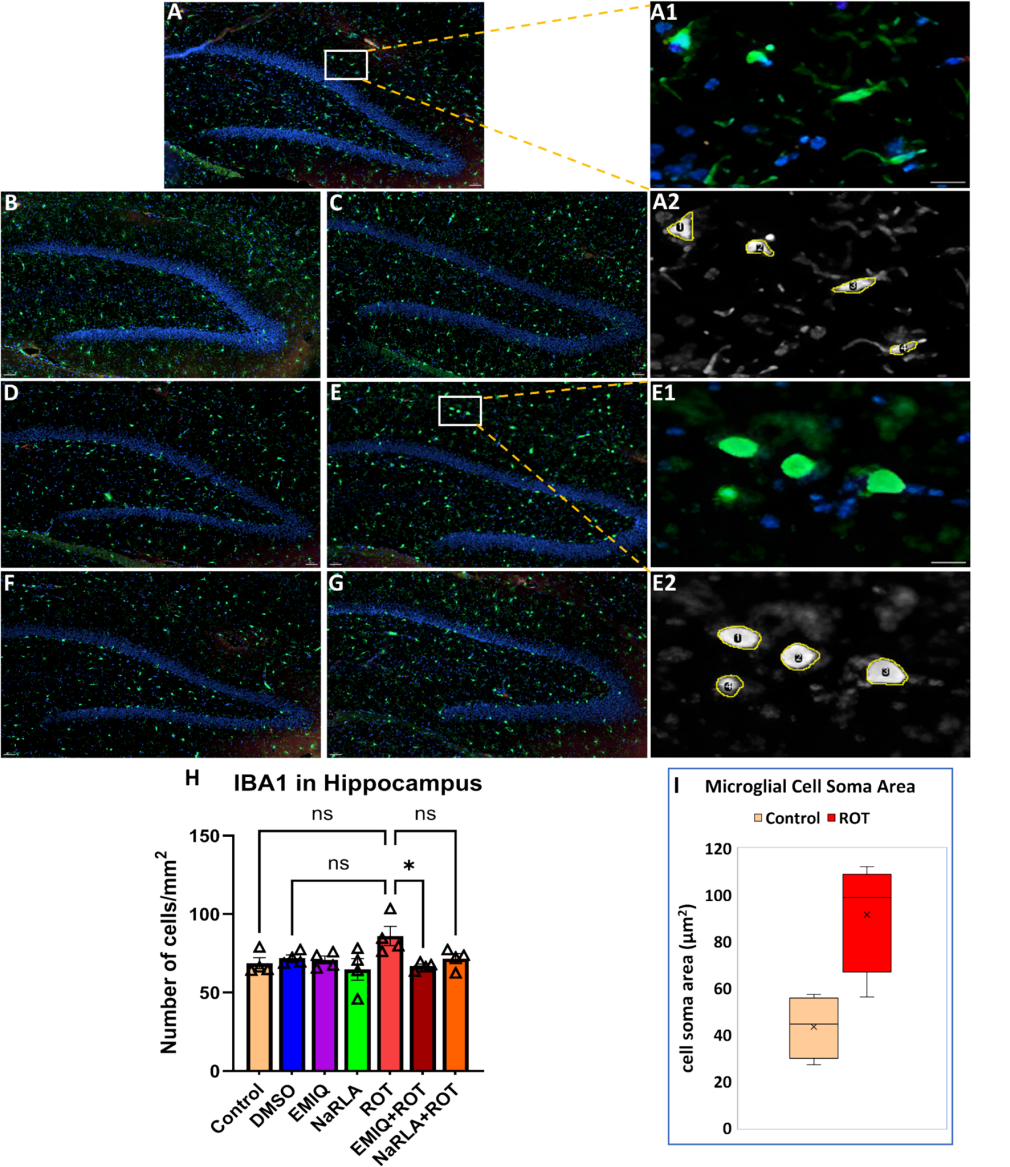
Effect of EMIQ and NaRLA on IBA1 positive microglia in the hippocampus of ROT-treated rats. Representative immunofluorescence photomicrographs of IBA1^+^ microglia in the hippocampus of normal control group (**A**), DMSO group (**B**), EMIQ group (**C**), NaRLA group (**D**), ROT group (**E**), EMIQ + ROT group (**F**), and NaRLA + ROT group (**G**). Scale bar = 50 µm. The highlighted areas show higher-magnification images of **A** and **E**, presented as images A1 and A2 (normal control) and E1 and E2 (ROT). These images illustrate microglial morphological differences (A1, E1) and Fiji-processed images of the cell soma area (A2, E2). Scale bar = 10 µm. A quantitative analysis of the number of IBA1^+^ microglia in the hippocampus is provided for all groups (H). Panel I shows the quantitative analysis of microglial area of cell soma (µm.^2^). Blue = DAPI, green = IBA1. Data presented as mean ± SEM (n = 4 rats/group and four sections per animal). (ANOVA with Tukey’s post hoc test; *P < 0.05, ns: no significance)

Regarding the number of microglial cells, rats administered DMSO, EMIQ or NaRLA revealed a non-significant change in the number of microglial cells/mm^2^ within the striatum (control: 64.64 ± 1.50, DMSO: 66.47 ± 4.99, EMIQ: 54.62 ± 2.60, NaRLA: 51.84 ± 3.81), SNpc (control: 76.46 ± 5.92, DMSO: 78.18 ± 2.37, EMIQ: 76.01 ± 5.40, NaRLA: 72.08 ± 4.82), cortex (control: 50.28 ± 2.78, DMSO: 59.43 ± 2.91, EMIQ: 55.48 ± 3.96, NaRLA: 48.24 ± 3.52), and hippocampus (control: 68.67 ± 3.51, DMSO: 72.04 ± 1.96, EMIQ: 70.79 ± 2.48, NaRLA: 64.81 ± 6.91) (Fig. 4, 5, 6 and 7). Highly significant increases in microglial cell density have been observed in the striatum, SNpc and cortex of rats treated with ROT compared to the normal control and DMSO groups (striatum: 110.30 ± 3.33, SNpc: 109.00 ± 8.23, cortex: 96.37 ± 10.03). Although the increase in microglia was not statistically significant in the hippocampus (86.06 ± 6.07), the morphology of microglia in the ROT-treated group was apparently distinctive. In a healthy, inflamed-free brain, microglia have a "ramified" shape. This means that they have a small cell body with many thin, branching processes. These processes constantly survey the surrounding microenvironment (Fig. 7A–D). Particularly during inflammation, activated microglia transform from their resting ramified shape into a larger, amoeboid or blob-like form. This morphological shift is characterized by the retraction of their numerous fine, branched processes, allowing them to migrate to injury sites and perform their immune functions effectively, such as phagocytosis (Fig. 7E and E1). Comparatively, the EMIQ + ROT group showed a significant decrease in microglial cell density in the hippocampus and striatum regions (66.93 ± 1.25 and 65.19 ± 5.73, respectively), in contrast to the ROT group, whereas such a decrease was non-significant in cortex (71.52 ± 7.78) and SNpc (86.35 ± 4.75). These results suggest that EMIQ was effective in reducing the degree of inflammation in the brain regions most affected by ROT (Fig. 4I). Similarly, treatment with NaRLA + ROT exhibited a significant decline in the number of microglial cells in the striatum (64.57 ± 2.20), while such decline was non-significant in the other investigated brain parts, including SNpc (100.70 ± 4.72), cortex (73.74 ± 5.09), and hippocampus (71.65 ± 3.17), compared to the ROT group. The Figs. 4, 5, 6 and 7 display highly representative immunofluorescence images. It seems likely that the anti-inflammatory effects of EMIQ and NaRLA are more pronounced in the striatum, a brain region that receives innervation from dopaminergic neurons. This region is important for motor control and is considered a hallmark of PD.

**Fig. 8:**
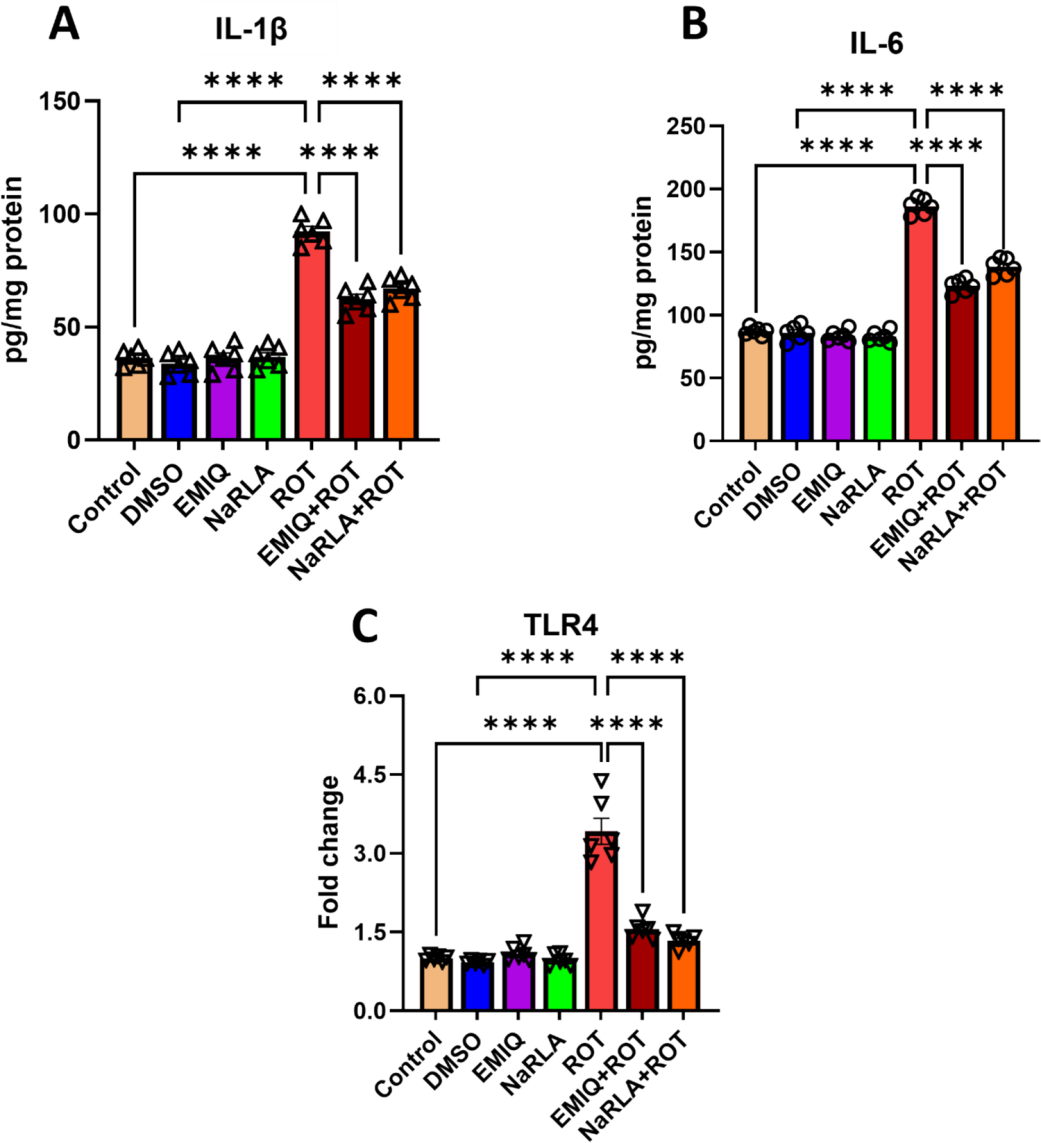
The impact of EMIQ and NaRLA administration on IL-1β (**A**), IL-6 (**B**), and TLR4 expression (**C**) in rats treated with rotenone. n = 6, Data are expressed as mean ± SEM, analyzed using one-way ANOVA with Tukey’s post hoc test for multiple comparisons, ****P < 0.0001

Furthermore, we investigated how exposure to ROT affected the secretion of pro-inflammatory cytokines (IL-1β and IL-6). Animals administered DMSO, EMIQ, or NaRLA showed a non-significant change in IL-1β (33.83 ± 1.96, 36.33 ± 2.29, 36.83 ± 1.91 pg/mg protein, respectively) and IL-6 levels (86.00 ± 2.44, 83.67 ± 1.80, 83.00 ± 1.79 pg/mg protein, respectively) in comparison with the normal control group (IL-1β: 36.33 ± 1.41; IL-6: 87.33 ± 1.28). Concentrations of IL-1β and IL-6 markedly elevated in ROT-injected rats (92.33 ± 2.28 and 186.33 ± 2.60 pg/mg protein, respectively) relative to the normal control or DMSO groups. Treatment with EMIQ or NaRLA drastically diminished the level of IL-1β in ROT-treated animals (62.33 ± 2.23 and 67.00 ± 2.02 pg/mg protein, respectively) and IL-6 concentrations (123.17 ± 2.23 and 138.50 ± 2.72 pg/mg protein, respectively) compared to rats treated with ROT alone (Fig. 8A and B). Furthermore, the vehicle group (DMSO), as well as the EMIQ and NaRLA groups, did not exhibit any significant changes in TLR4 expression relative to the normal control group. Real-time PCR results showed a dramatic four-fold increase in TLR4 gene expression in the striatum of rats exposed to ROT when compared to normal control or DMSO groups. Rats treated with EMIQ + ROT or NaRLA + ROT showed a significant decline in TLR4 expression compared to ROT-treated rats (Fig. 8C).

## Discussion

PD is a progressive, chronic pathological condition, which is positioned as the second most prevalent among neurodegenerative disorders. The condition is manifested by the gradual deterioration of dopaminergic neurons located within SNpc. Motor deficits, including bradykinesia, tremors, and rigidity are associated with a shortage of DA in the striatum, which is an outcome of the nigral neurons damage [41]. The disabling effects of PD can be replicated using various animal models such as MPTP, 6-hydroxydopamine and ROT. These models are also widely used in preclinical studies to develop new drugs for treating PD. ROT as a mitochondrial complex I inhibitor, is a valuable research model for studying the key pathological features of PD because it uniquely mimics mitochondrial dysfunction. This study aimed to investigate region-specific vulnerability in the striatum compared with the cortex and hippocampus, to characterize associated inflammatory responses, and to evaluate the neuroameliorative effects of EMIQ and NaRLA in a ROT-induced rat model of PD. In the present study, ROT exposure induced motor deficits alongside dopaminergic impairment and neuroinflammatory alterations. Treatment with EMIQ or NaRLA effectively mitigated these changes, highlighting their neuroprotective and anti-inflammatory potential.

Regarding behavioral tests, hanging and open field tests demonstrated locomotor dysfunction in rats treated with ROT, represented in little number of squares passed, low rearing, long freezing time, and short hanging time. These findings are consistent with previous reports [42, 43]. Muscle coordination and motor performance were substantially enhanced by EMIQ and NaRLA. Quercetin demonstrated beneficial effects against ROT, as evidenced by significant improvements in grip strength, rearing behavior, and catalepsy performance [44, 45]. Moreover, Isoquercitrin improved behavioral outcomes in a subacute MPTP-induced PD model, including better rotarod performance and higher pole-climbing and suspension test scores [24]. In addition to its established antioxidant properties, EMIQ has been reported to support muscle function under conditions of stress, which may further contribute to the improvement of motor performance observed in the present ROT model [46]. A recent study revealed that the administration of ALA markedly improved behavioral outcomes. These included enhanced exploratory activity (rearing), and increased locomotor performance in the open field test [47]. Furthermore, ALA administration led to substantial increases in distance traveled, locomotor speed, and rearing frequency, accompanied by a reduction in immobility time compared with the ROT-treated group [30]. Due to its amphipathic nature, ALA can function in both aqueous and lipid environments. This property enables it to cross the blood brain barrier and exert effects in the cytoplasm, plasma membranes, and lipoproteins [48]. When administered in vivo, NaRLA is anticipated to exhibit enhanced efficacy in comparison to ALA due to its considerably higher water solubility and considerably more advantageous pharmacokinetic profile [29]. One of the possible protective mechanisms of ALA is the activation of PI3K/AKT pathway. This activation contributes to the attenuation of oxidative stress and inflammation, along with improvements in behavioral outcomes, thereby underscoring its multifaceted antioxidant action [30].

Nigrostriatal dopaminergic neurons depletion was the outcome of ROT treatment, as evidenced by a significant decline in striatal DA concentrations and depletion of TH^+^ dopaminergic fibers in striatum, and dopaminergic cells in SNpc. Previous research demonstrated the deleterious effect of ROT administration on DA concentration in striatum tissue and loss of SNpc TH-positive cells [20, 43, 49, 50]. ROT is known to cross the blood–brain barrier easily and cause neurotoxicity by inhibiting mitochondrial complex I, and in rats, ROT produces behavioral, neuropathological and biochemical symptoms that resemble PD in humans. Both complex I inhibition and oxidative stress are caused by MPTP and ROT and can exacerbate each other, which ultimately lead to the death of the dopaminergic neurons. This note also opens the door to the protective effect of antioxidants on the dopaminergic neurons. Of course, the importance of these mechanisms in causing the death of DA neurons depend on the route, dose of toxin exposure, duration as well as the animal species [51]. In the present study, the decrease in TH^+^ expression in SNpc and the significant decrease in TH^+^ fibers in striatum (about 50% decline compared to normal control) may be attributed to the progressive properties of ROT as a PD model which indicates that SNpc may not be the first affected part [52]. These alterations in dopaminergic markers likely underlie the motor deficits observed in ROT-treated animals. The reduction in DA levels in the striatum may lead to the neurodegeneration of axon terminals, which is the initial site affected in PD models [53].

Rats cotreated with EMIQ + ROT showed a significant improvement in striatal DA level, accompanied by a notable improvement in TH-positive cells of SNpc and striata compared to rats treated with ROT alone. So far, EMIQ specifically has not been tested in ROT PD models. However, related compounds including isoquercitrin and quercetin in murine PD models show consistent antioxidant, anti-inflammatory, and anti-apoptotic effects. Karuppagounder et al. (2013) reported that treatment with quercetin significantly mitigated ROT-induced striatal DA depletion in rats through upregulating complex I activity [54]. Administering 100 and 200 mg/kg of quercetin resulted in a notable elevation in DA levels in mice treated with MPTP-induced PD [55]. Moreover, pretreatment with isoquercitrin showed a significant recovery in dopaminergic neurons of striatum and SNpc of MPTP-treated mice [24]. In a rat model, it was demonstrated that EMIQ treatment (at 0.25% or 0.5% in the diet) initiated during late pregnancy suppressed lipopolysaccharides (LPS)-induced neuroinflammation in offspring. The detailed immunochemical analyses revealed that EMIQ ameliorates the proinflammatory and oxidative responses caused by LPS that lead to brain damage [56]. Thus, due to its higher bioavailability and anti-inflammatory and antioxidant properties, EMIQ may be able to provide neuroprotection to dopaminergic neurons in the striatum and SNpc in rat model of PD.

Similarly, oral administration of NaRLA in ROT-treated rats resulted in a substantial elevation of striatal DA concentration, alongside a highly significant enhancement in TH^+^ fibers within the striata together with a notable rise in TH^+^ cells in the SNpc, although the latter was non-significant compared to rats treated with ROT alone. In accordance, Zaitone et al. (2012), examined the impact of ALA on rats treated with ROT, and their results demonstrated a significant improvement in the striatal DA level relative to ROT group [57]. In addition, ALA has the propensity to ameliorate mitochondrial respiratory chain enzymes dysfunctions and oxidative stress in rat Huntington’s disease model [58]. Recently, in ROT mouse model, ALA (100 mg/kg, i.p.) showed neuroprotective and anti-inflammatory effects, linked to PI3K/AKT pathway activation and improved behavior [30]. Accordingly, the preservation of DA levels and TH immunoreactivity by EMIQ and NaRLA may explain the improvement in motor behavior observed in treated animals.

Following brain injury, microglia activate, proliferate, and release toxic substances and proinflammatory cytokines, contributing to dopaminergic neuron death [11, 12, 14]. Consistent with this mechanism, neuroinflammation has long been implicated in the pathogenesis of PD, as evidenced by the abundant presence of activated microglia in the SNpc and striatum of both PD patients and experimental animal models [59]. It is also well established that ROT can trigger an inflammatory response in vivo and/or activate microglia through proinflammatory pathways. IBA1 immunoreactivity showed that microglial responses were regionally heterogeneous after ROT administration. Qualitative analyses revealed a significant increase in microglial staining density within the substantia nigra and striatum but less pronounced in cortex compared with vehicle-treated rats [60], however in a mouse study the increase in microglial number was observed in substantia nigra but not in striatum [61]. Consistent with a previous study [62], we found that the SNpc, hippocampus and striatum contain a greater number of IBA1 microglia than the cortex under normal control conditions. The high number of microglia in the substantia nigra compared with other brain regions may explain how inflammation can cause the degeneration of the nigrostriatal pathway in PD. Taken together, the results of our study showed that ROT causes significant increases in the number of microglia in specific brain regions, such as the SNpc, striatum and cortex. Nevertheless, the difference did not achieve statistical significance in the hippocampus, where a significant increase in microglial cell soma was observed in our preliminary analysis. These findings highlight the non-uniform nature of microglial activation in ROT models of PD, affecting both the degree and phenotype of microglial responses across brain regions. Further studies are needed to clarify the association between microglial number and activation state as well as morphologic activation without proliferation.

Empirical evidence from this study indicates that ROT not only was able to increase the numbers of microglia in several brain regions but also was able to upregulate the expression of key mediators of inflammation IL-1β and its main downstream target IL-6 as well as TLR4, which play a major role of inflammation in the PD [15], thereby intensifying neuroinflammatory processes. To confirm, TLR4 knockout lowered the number of activated microglial cells and protected against SNpc dopaminergic degeneration, demonstrating that TLR4 contributes to neuroinflammation [63]. In view of their pivotal functions in the process of inflammation, the targeting of both IL-1β and IL-6 has been investigated as a therapeutic approach to decrease inflammation via EMIQ and NaRLA administration. Both compounds inhibited the excessive microglia activation in brain of ROT-treated rats, thus reducing the expression of TLR4, IL-1β and IL-6 in this study. It is worthy of note that quercetin reduced the production of IL-1β and monocyte chemoattractant protein-1, in addition to decreasing microglial activation and inflammation-induced neuronal death in brain tissue [64, 65]. The anti-inflammatory effects of isoquercitrin may be associated with its suppression of nuclear factor kappa B (NF-κB) nuclear translocation, which controls the expression of IL-6, and IL-1β in an NF-κB-dependent manner [66]. Indeed, previous studies have shown that the plasma concentration of conjugated quercetin metabolites reaches a maximum level approximately 1.5 h after EMIQ intake and substantial amount of intact isoquercitrin have been detected in rat brain [67]. Similarly, ALA treatment significantly decreased IL-6 and TNF-α levels in LPS-treated rats [68]. LPS-induced NF-κB activity was suppressed by lipoic acid, leading to a decline in IL-6 production via protein kinase A activation, which subsequently inhibits TLR4 [69]. In addition, ALA treatment resulted in reduced microglial activation IBA1⁺ in MPTP-induced mice compared to untreated controls, indicating its potential to mitigate MPTP-induced neuroinflammation in the SNpc and spinal cord [70]. The mechanisms behind this include antioxidant support, mitochondrial stabilization, and anti-inflammatory/anti-apoptotic signaling [71]. While results are preclinical and protocol-dependent, the relationship is broadly beneficial and/or protective in rats. To the best of our knowledge, this is the first time to explore the effect of NaRLA, the sodium salt of ALA on ROT rat model of PD. Accordingly, we hypothesize that the inhibitory effects of EMIQ and NaRLA on TLR4 cascade and inflammatory response could alleviate blood brain barrier damage and the subsequent entry of ROT and its toxic metabolite into the brain, thereby reducing neuroinflammation.

Taking together, both EMIQ and NaRLA are innovative substances with high bioavailability and stability. Due to its human safe, EMIQ is currently used as a food additive and a constituent of dietary supplements [72]. Using of NaRLA solved the absorption and polymerization problem of ALA and maintaining antioxidant functions. Given that EMIQ and NaRLA are derivatives of quercetin and α-lipoic acid, respectively, they are likely to share similar neuroprotective pathways; however, further comparative studies are required to confirm these mechanisms in the context of increased bioavailability and solubility. It is necessary that further clinical trials with human subjects are carried out to provide more detailed information about the possibilities and limitations of the potential health effects of both substances.

## Conclusion

The findings demonstrated that EMIQ and NaRLA provide protection in rats against ROT induced PD model via their neuroameliorative effects. Both compounds alleviated ROT-induced behavioral impairments, improved TH positive neurons and reduced neuroinflammation. Importantly, the increase in microglial density was selective, meaning that there is a dissociation between microglial number and activation state, and these factors are brain region-specific suggesting that some brain regions may show strong activation without proliferation. Collectively, these results imply that EMIQ and NaRLA may be vital as adjuvant treatment and multimodal support for PD.

## Supplementary Information

Below is the link to the electronic supplementary material.

Supplementary file1 (PPTX 30827 KB)

## Data Availability

The data that support the findings of this study are available from the corresponding author upon reasonable request.
