# Supplementary material for: The Neuroameliorative Effects of Enzymatically Modified Isoquercitrin and Sodium R-lipoate on the Rotenone rat Model of Parkinson’s Disease
Source: Neurochem Res. 2026 Mar 25;51(2):120. doi: 10.1007/s11064-026-04715-9 (PMC13018002; doi:10.1007/s11064-026-04715-9)
Supplement: Supplementary file 1 — Supplementary file1 (PPTX 30827 KB) [file 11064_2026_4715_MOESM1_ESM.pptx]

## Slide 1
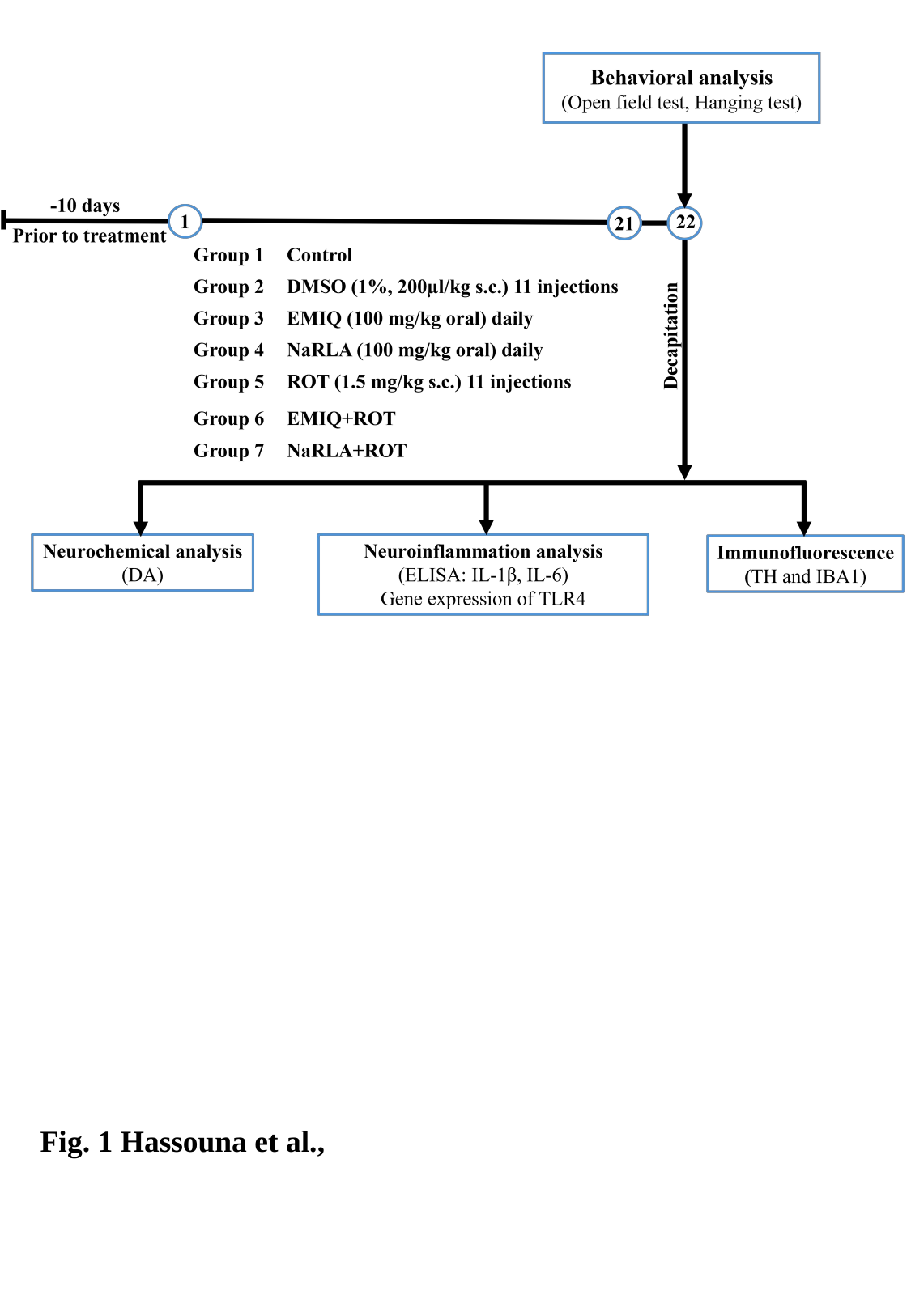

Fig. 1 Hassouna et al.,

## Slide 2
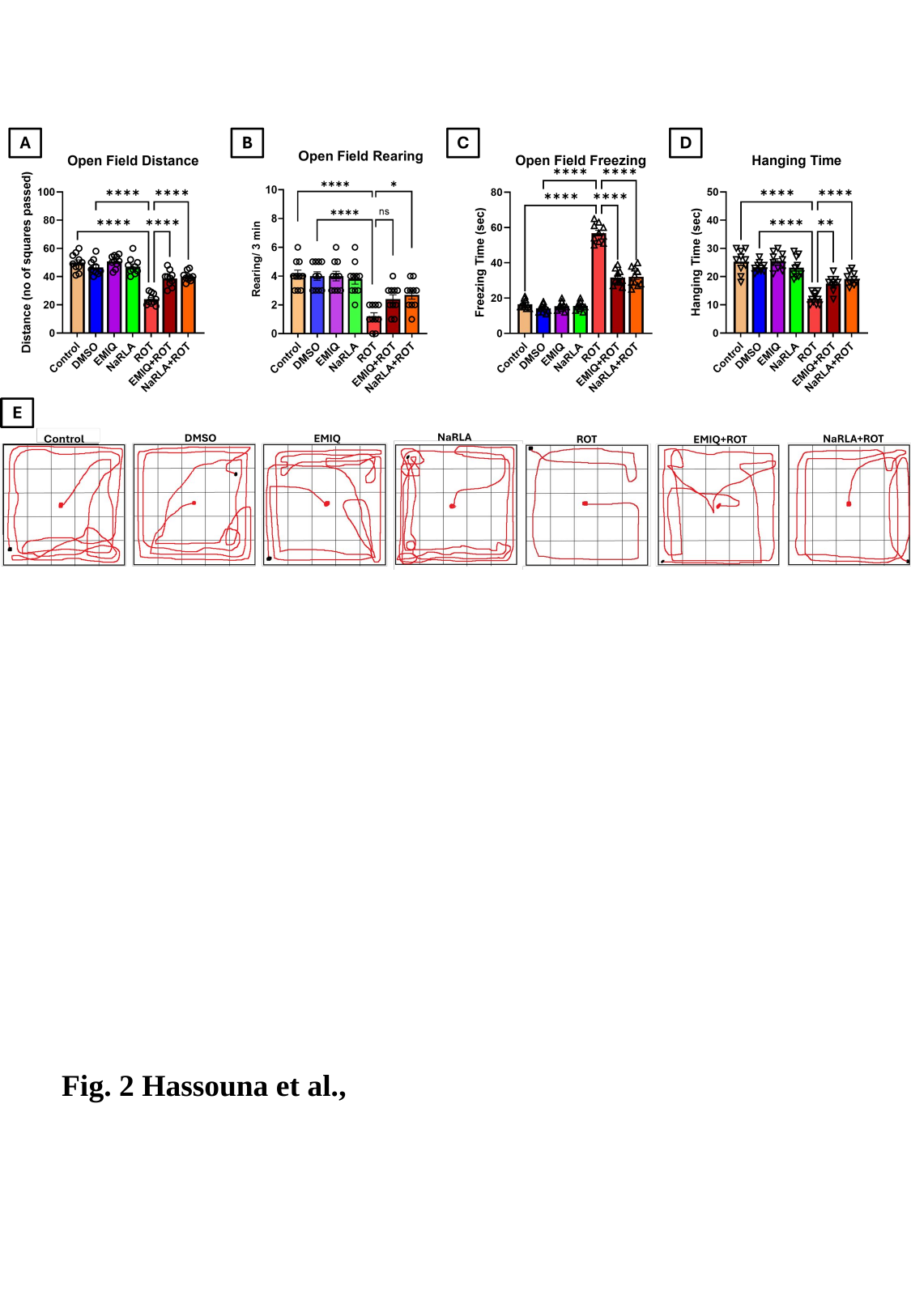

Fig. 2 Hassouna et al.,

## Slide 3
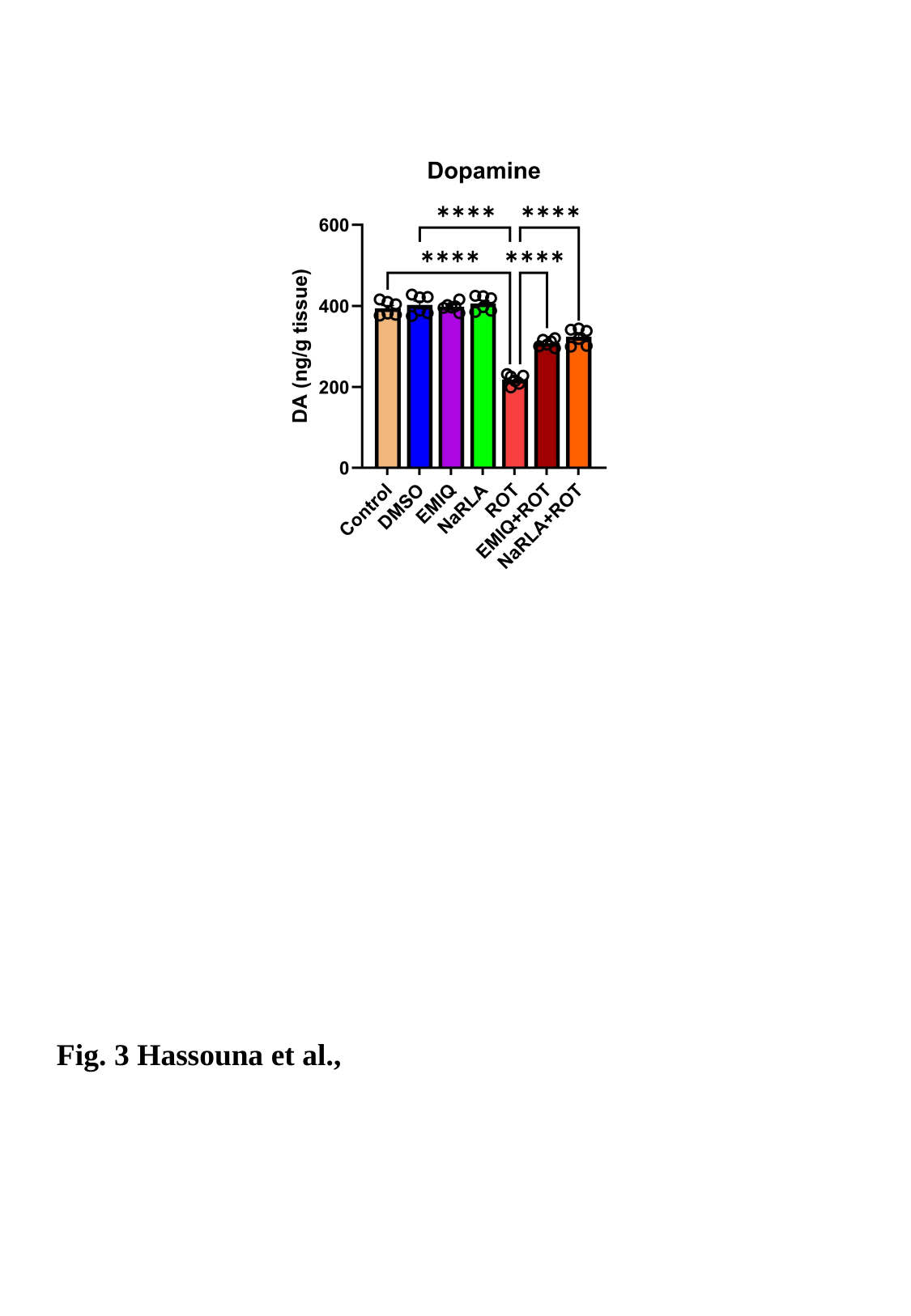

Fig. 3 Hassouna et al.,

## Slide 4
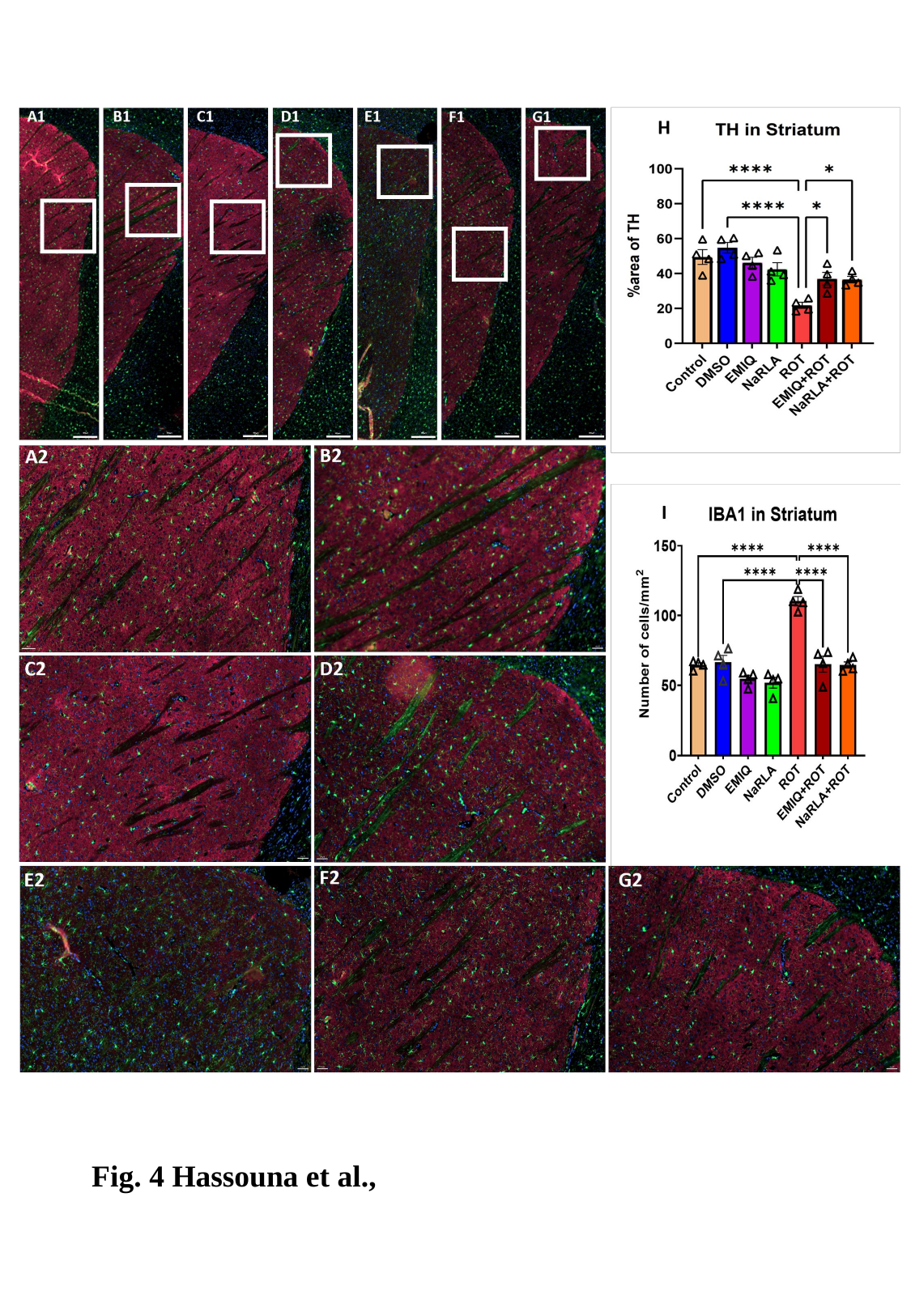

Fig. 4 Hassouna et al.,

## Slide 5
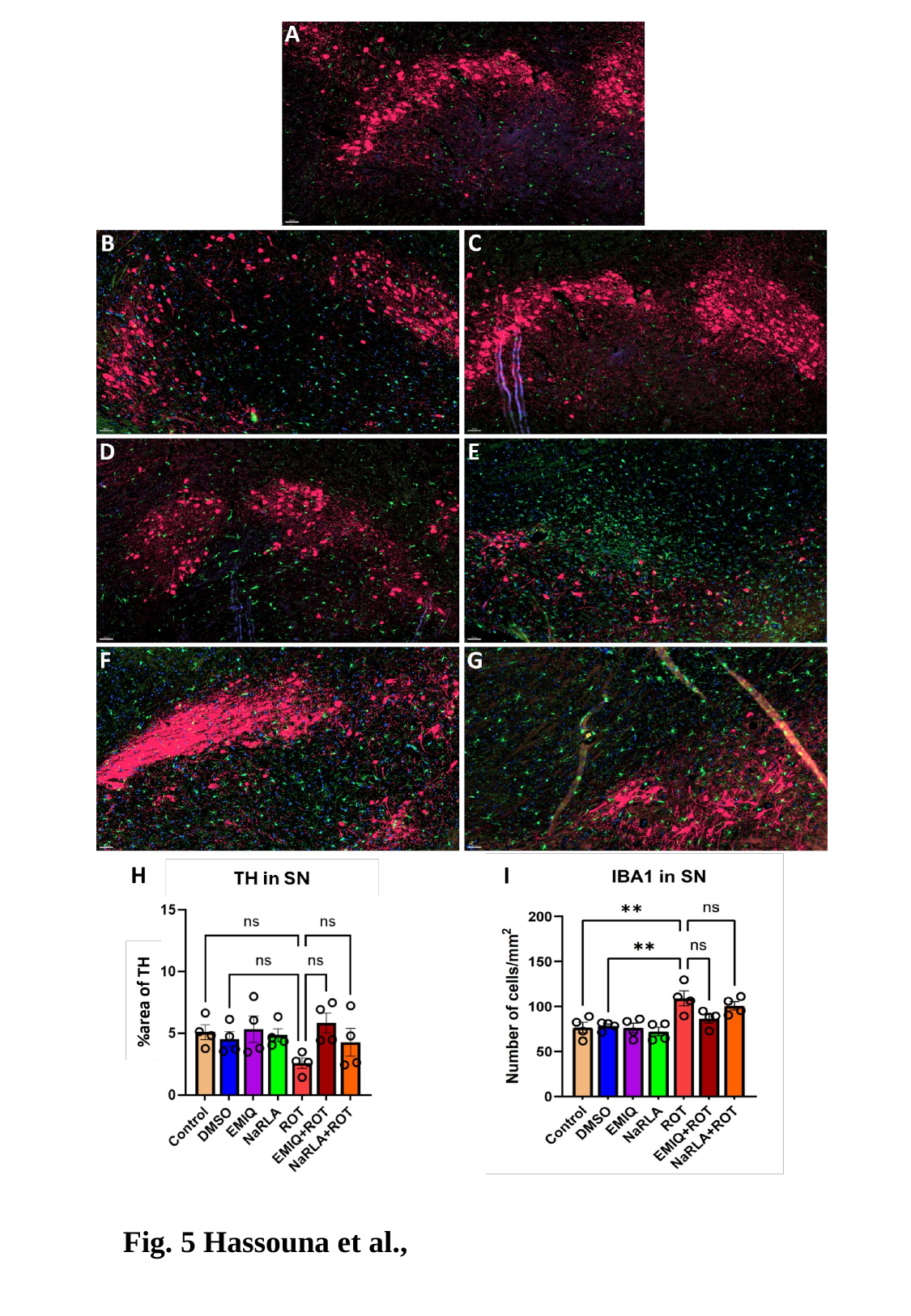

Fig. 5 Hassouna et al.,

## Slide 6
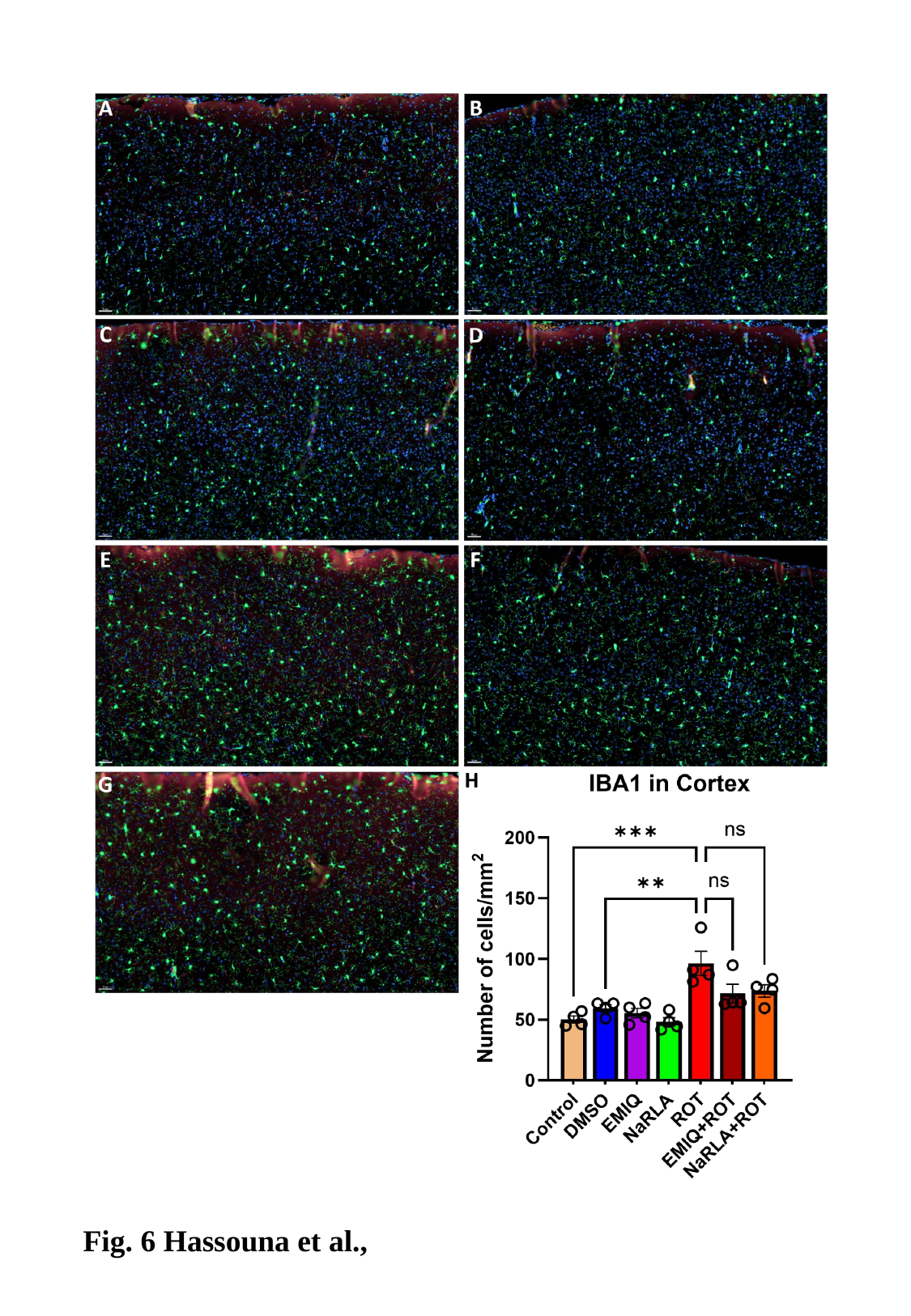

Fig. 6 Hassouna et al.,

## Slide 7
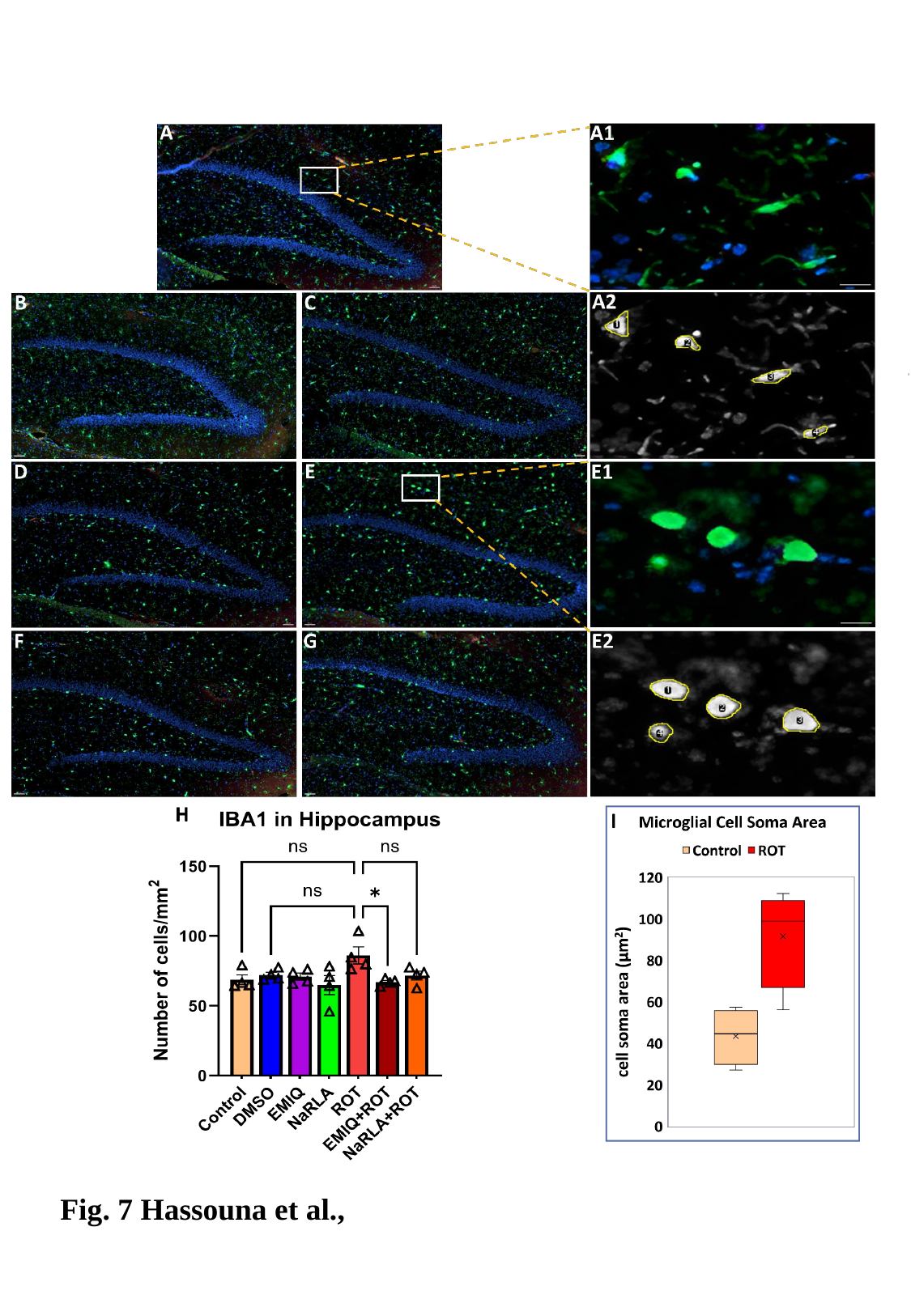

Fig. 7 Hassouna et al.,

## Slide 8
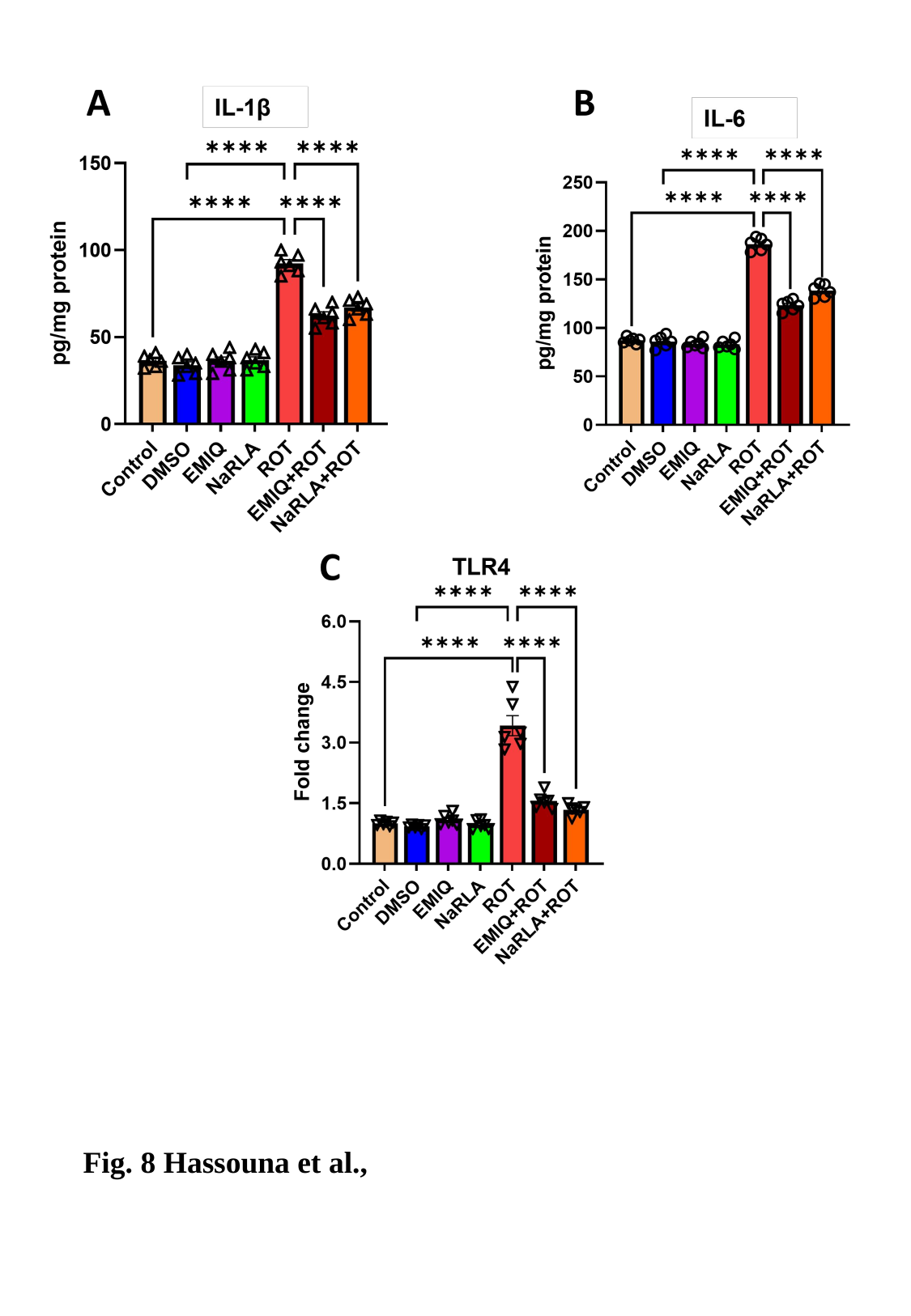

Fig. 8 Hassouna et al.,
